# Stigma of mental illness in the gypsy ethnic group

**DOI:** 10.1192/j.eurpsy.2023.1438

**Published:** 2023-07-19

**Authors:** M. V. López Rodrigo, A. Osca Oliver, M. Palomo Monge, V. Ros Fons, Y. D´Hiver Cantalejo

**Affiliations:** Psiquiatría, Hospital Nuestra Señora del Prado, Talavera de la Reina, Spain

## Abstract

**Introduction:**

The Roma population constitutes the largest ethnic minority in Spain (more than 2% of the population), with our country having the third largest total population of Roma in the world. The concept of health and disease varies with the sociocultural context. It is important to know the cultural characteristics to exercise good clinical practice. The stigma surrounding mental illness is widely known, and is even stronger in the Roma community, leading to marginalization and shame.

**Objectives:**

We present a case of a gypsy woman misdiagnosed from the age of 8 with hebephrenic schizophrenia.

**Methods:**

Patient frequents the emergency department with symptoms of predominantly anxiety, including episodes of psychomotor agitation, self-harm, verbalization of visual hallucinations of a mystical-religious nature. In treatment with antipsychotics since diagnosis, with no therapeutic adherence. It is observed during all the episodes how the anxiolytic treatment, even, sometimes, the verbal restraint, make the symptoms subside. Psychotic symptoms over the years are ruled out.

**Results:**

Due to the diagnosis, this patient has been relegated from the gypsy community, she has not married or had children (an important milestone in gypsy culture), this has generated an exponential increase in anxiety symptoms and home problems.

**Conclusions:**

It is important to know the cultural traits to which the patients we treat in consultation belong, and how the disease can affect their lives, and a simple diagnosis can be a source of greater anxiety.

**Disclosure of Interest:**

None Declared

